# Editorial: Pediatric anesthesia and surgery: prophylaxis, managements, and rehabilitation of short-term and long-term complications of CNS during perioperative period

**DOI:** 10.3389/fmed.2023.1342556

**Published:** 2023-12-07

**Authors:** Jie Zeng, Zen Fan, Cong Yu

**Affiliations:** ^1^Department of Anesthesiology, Stomatological Hospital of Chongqing Medical University, Chongqing, China; ^2^Chongqing Key Laboratory of Oral Diseases and Biomedical Sciences, Chongqing, China; ^3^Chongqing Municipal Key Laboratory of Oral Biomedical Engineering of Higher Education, Chongqing, China; ^4^State Key Laboratory of Military Stomatology, Department of Anesthesiology, School of Stomatology, Fourth Military Medical University, Xi'an, China

**Keywords:** pediatric anesthesia, complications of CNS, HFNO, remimazolam, pain management

The issue of perioperative central nervous system complications in pediatric patients is attracting heightened awareness. The current treatment outcomes are not ideal, due to inadequate monitoring and intervention techniques. To enhance our grasp of the clinical presentation and underlying mechanisms, we have curated a collection of seven studies under the Research Topic “*Pediatric anesthesia and surgery: prophylaxis, managements, and rehabilitation of short-term and long-term complications of CNS during perioperative period*”. Four articles on adopting new anesthesia techniques and monitoring methods to improve central nervous system complications in children are included, as well as two studies on the effects of new anesthetic drugs or dosages, and one comprehensive review. These studies explore innovative management approaches within anesthesia during the perioperative period. They address the occurrence and recovery of central nervous system complications from multiple angles, including enhancing perioperative pain management and optimizing medication regimens.

In the original article by Liu et al., the application of a new ventilation technique, high-flow nasal oxygen (STRIVE Hi), in laryngotracheal surgery for infants and children was studied. A sufficient oxygen supply is beneficial for brain protection during such surgeries, conducted under spontaneous breathing without intubation. The authors suggest that STRIVE Hi positively affects children undergoing non-intubated laryngeal surgery under spontaneous ventilation, reducing the hazards associated with high oxygen concentrations compared to using a relatively low 70% oxygen concentration.

Yang et al. assessed the impact of internal jugular vein cannulation during robotic-assisted laparoscopic surgeries on intracranial pressure and postoperative delirium by measuring the optic nerve sheath diameter. The required Trendelenburg position and CO_2_ pneumoperitoneum for robotic-assisted surgeries significantly affect cerebral blood flow and intracranial pressure, affecting perioperative cognitive functions. The study suggests that internal jugular vein cannulation may not be the preferred method for such surgeries due to its association with increased intracranial pressure, venous reflux, and delayed awakening.

Bove's study explored the efficacy of ultrasound-guided dorsal penile nerve block (DPNB) for circumcision surgeries. It is well-known that nerve blocks combined with moderate sedation can provide good anesthesia, and effective pain relief is essential to reduce neurological complications. The author included 70 patients and found that ultrasound-guided DPNB combined with sedation and spontaneous breathing is a timesaving, opioid-sparing, safe, and effective strategy for managing perioperative pain in child circumcision surgeries.

In the research by Wu et al., a WeChat mini-program–based perioperative rehabilitation mobile health management platform was developed, which allows online follow-up with cancer surgery patients regarding their recovery and nutritional status. This trend is being embraced globally as electronic medical services become increasingly prevalent. Further investigation revealed that most patients and healthcare workers believe this method can improve the current level of medical services and knowledge of nutritional health, promoting communication between healthcare workers and patients.

Long et al. identified the ED 95 of intranasal midazolam for alleviating preoperative anxiety in children. Midazolam, a new drug used clinically, is beneficial for managing perioperative central nervous system complications by relieving preoperative anxiety. After a biased coin design trial in 80 patients, the authors concluded that the ED 95 of a single intranasal dose of midazolam for reducing preoperative anxiety in early childhood children and preschool children is 1.57 and 1.09 mg·kg^−1^, respectively.

Chen et al. conducted a network meta-analysis comparing the effectiveness and safety of parecoxib and ibuprofen ester for perioperative analgesia in children. This study also focused on perioperative pain management, comparing the pros and cons of multimodal analgesia plans using commonly used NSAIDs. The authors believe ibuprofen ester is most effective in reducing pain scores at 2, 4, and 12 h post-surgery. Parecoxib has advantages in reducing pain scores at 0, 0.5, 1, 6, 8, and 24 h post-surgery, as well as the overall incidence of adverse events and postoperative nausea and vomiting.

Additionally, in a review by Gao and Wu, the current progress of procedural sedation in pediatric dentistry was summarized, from the definition of procedural sedation to the current mainstream methods and commonly used drugs, with a particular emphasis on patient safety during sedation.

In summary, this Research Topic “*Pediatric anesthesia and surgery: prophylaxis, managements, and rehabilitation of short-term and long-term complications of CNS during perioperative period*” for Frontiers in Medicine (intensive care medicine and anesthesiology) outlines the current progress and ongoing research directions in the management and rehabilitation of pediatric perioperative central nervous system complications. The discussed research emphasizes the work required to implement effective interventions and prevention strategies, with various new management methods and drugs highlighting the importance of research. We also strive to provide more opportunities for researchers to present their work.

## Author contributions

JZ: Writing—original draft. ZF: Writing—original draft. CY: Conceptualization, Writing—review & editing.

